# Supplementary material for: Gapless superconductivity in Nb thin films probed by terahertz spectroscopy
Source: Nat Commun. 2023 May 12;14:2737. doi: 10.1038/s41467-023-38422-8 (PMC10182076; doi:10.1038/s41467-023-38422-8)
Supplement: Supplementary file 1 — Supplementary Information [file 41467_2023_38422_MOESM1_ESM.pdf]

Supplementary Information for "Gapless superconductivity in Nb thin films probed by terahertz spectroscopy"

Ji Eun Lee[1,*], Joonyoung Choi[2,*], Taek Sun Jung[1], Jong Hyuk Kim[1], Young Jai Choi[1], Kyung Ik Sim[3,4,†], Younjung Jo[2,†], and Jae Hoon Kim[1,†]

Affiliations

[1]Department of Physics, Yonsei University, Seoul 03722, Republic of Korea

[2]Department of Physics, Kyungpook National University, Daegu 41566, Republic of Korea

[3]Center for Integrated Nanostructure Physics, Institute for Basic Science, Suwon 16419, Republic of Korea

[4]Sungkyunkwan University, Suwon 16419, Republic of Korea

*These authors contributed equally to this work

[†]Corresponding author. Email: simki323@gmail.com, jophy@knu.ac.kr, super@yonsei.ac.kr

Contents:

Supplementary Fig. 1: Effect of an in-plane magnetic field on the transport properties of thin-film Nb.

Supplementary Fig. 2: Terahertz spectra of superconducting Nb at various temperatures in the absence of an external magnetic field.

Supplementary Fig. 3: Optical conductivity of a superconductor according to the Skalski—Betbeder-Maribet—Weiss Theory.

Supplementary Fig. 4: Additional data analysis on the in-plane magnetic field effect on the superconducting parameters of Nb.

Supplementary Fig. 5: Density of states of a pair-broken superconductor under an external in-plane magnetic field.

Supplementary Fig. 6: In-plane magnetic field dependence of the London penetration depth

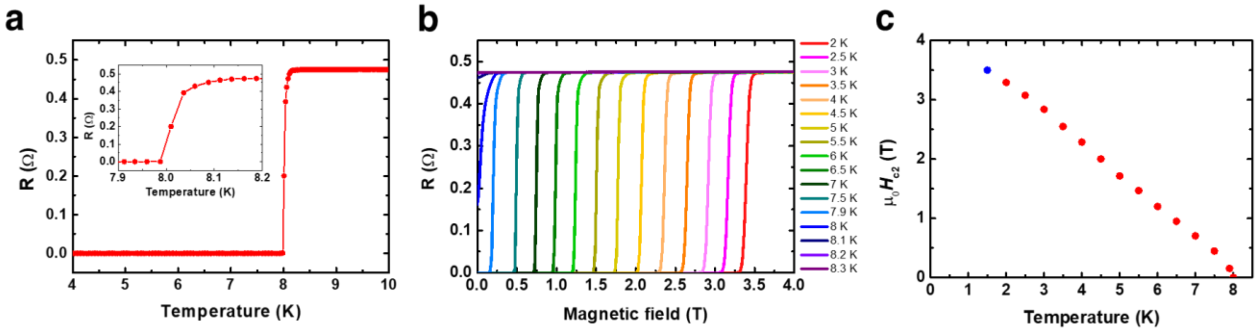

Supplementary Fig. 1 | Effect of an in-plane magnetic field on the transport properties of thin-film Nb.

a, Temperature dependence of the resistance of thin-film Nb (T_c=8 K). Inset: Magnified view near the superconducting transition in the temperature range of 7.9 K to 8.2 K. **b,** Superconducting transitions of thin-film Nb under various in-plane magnetic fields for the temperature range of 2 K to 8.3 K. **c,** Temperature dependence of the in-plane upper critical field $\mu_0 H_{c2}$ of thin-film Nb as extracted from **b** (red circles). The value of $\mu_0 H_{c2}$ is estimated to be 3.5 T at 1.5 K from our terahertz data (indicated by the blue circle).

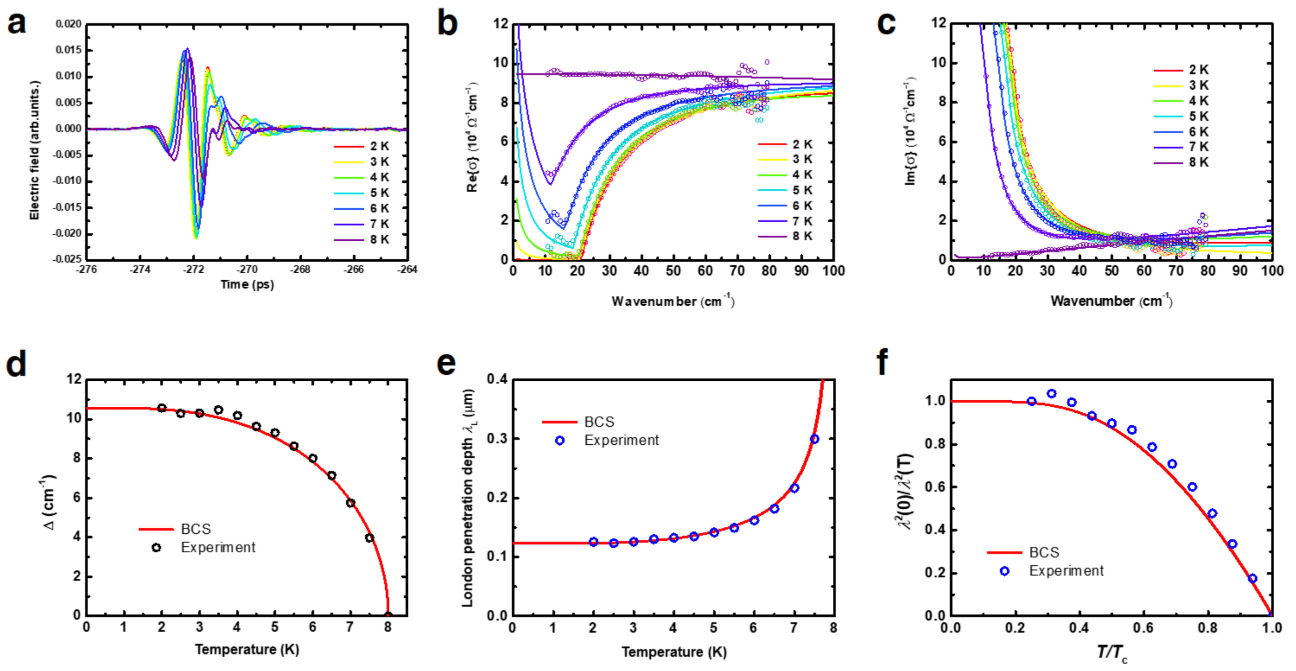

Supplementary Fig. 2 | Terahertz spectra of superconducting Nb at various temperatures in the absence of an external magnetic field.

a, Terahertz electric field waveforms transmitted through a Nb thin film over the temperature range from 2 K to 8 K. **b, c,** Real and imaginary parts of the optical conductivity of Nb (open circles) and the Zimmermann fits (solid lines). In the real part of the optical conductivity at the lowest temperature, the kink corresponds to twice the maximum superconducting gap. **d,** Temperature dependence of the superconducting gap of Nb. The open circles are experimental gap values obtained from the Zimmermann fits of the optical conductivity, and the solid line is the weak-coupling BCS fit. **e,** London penetration depth of Nb. The open circles are experimental London penetration depth values obtained from the imaginary part of the optical conductivity by using the relation $\sigma_2(\omega) = c^2/4\pi\lambda_{\mathrm{L}}^2\omega$ at $\omega/2\pi c =$ 0.97 cm^{-1}, and the solid line is the weak-coupling BCS fit. **f,** Inverse squared penetration depth normalized the zero-temperature value. This is the same as the superfluid density normalized to the zero-temperature value. The solid line is the BCS theory fit.

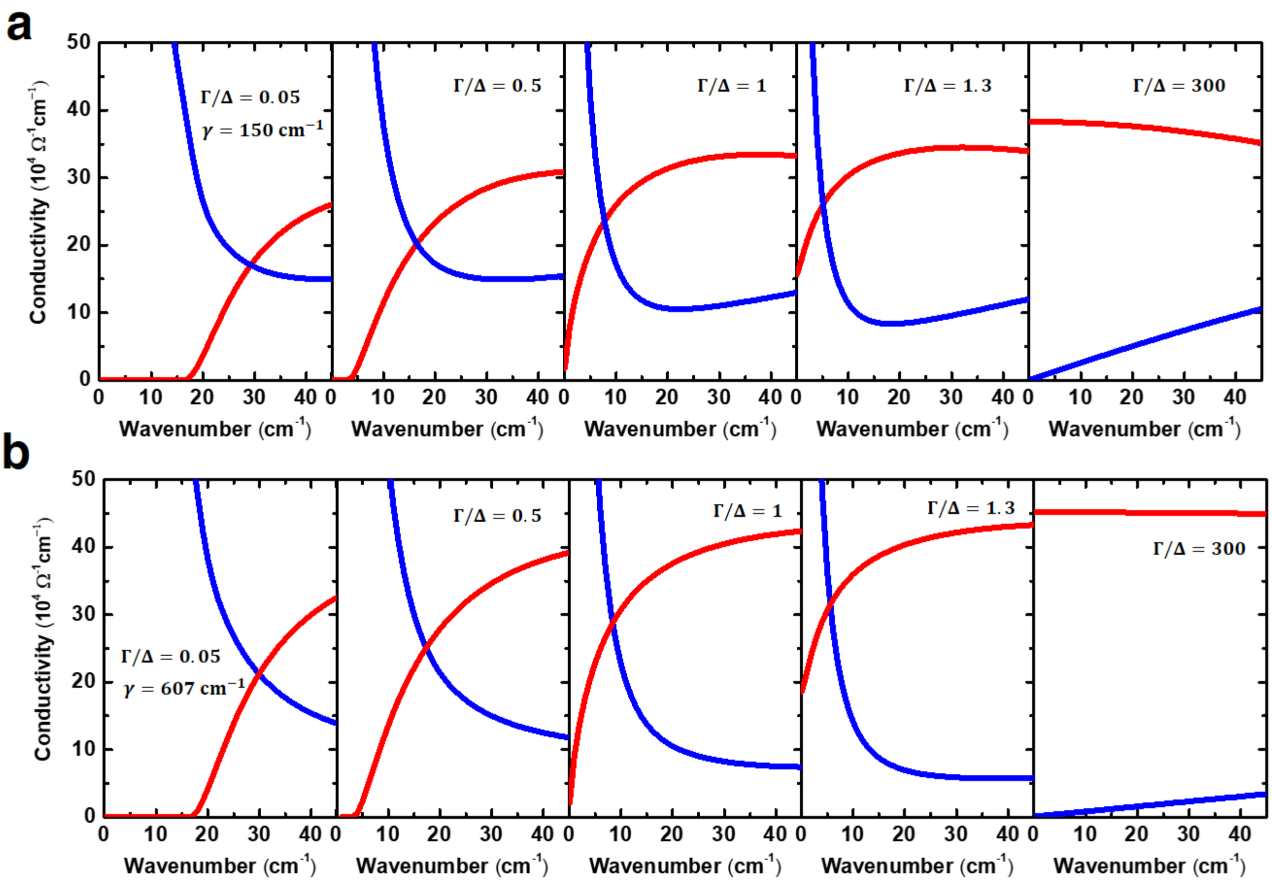

Supplementary Fig. 3 | Optical conductivity of a superconductor according to the Skalski—Betbeder-Maribet—Weiss Theory.

Real (σ_1, red) and imaginary (σ_2, blue) parts of the optical conductivity of a superconductor in the superconducting state (σ_s) as predicted by the pair-breaking theory Skalski, Betbeder-Matibet, and Weiss for various values of $\Gamma(H)/\Delta(H)$ at T=0 K. **a,** for a scattering rate of $\gamma = 150\ \mathrm{cm}^{-1}$ **b,** for $\gamma = 607\ \mathrm{cm}^{-1}$ applicable to our Nb thin films.

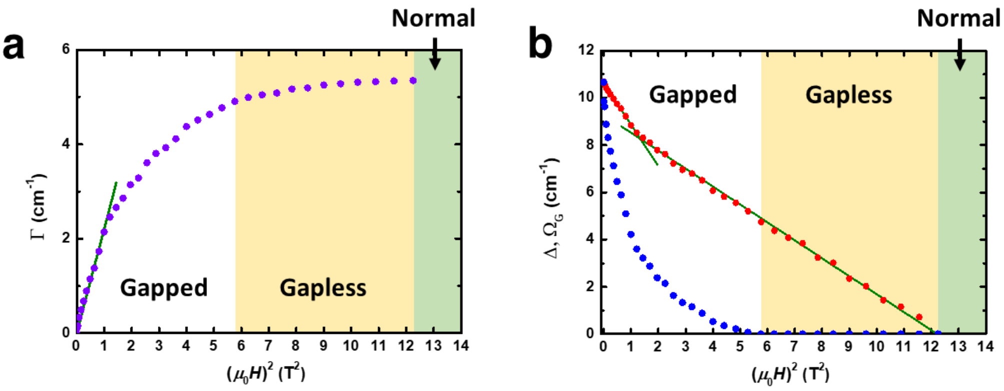

Supplementary Fig. 4 | Additional data analysis on the in-plane magnetic field effect on the superconducting parameters of Nb.

a, The relationship between the pair-breaking parameter Γ and the squared magnetic field H^2. The white, yellow, and green shaded regions indicate the gapped superconducting state, the gapless superconducting state, and the normal state. The green line is a linear fit representing a quadratic dependence on the magnetic field. **b**, The spectroscopic gap Ω_G (blue circles) and the superconducting order parameter Δ (red circles) as functions of the squared magnetic field H^2. The two green lines are linear fits representing the quadratic dependences of the order parameter on the magnetic field.

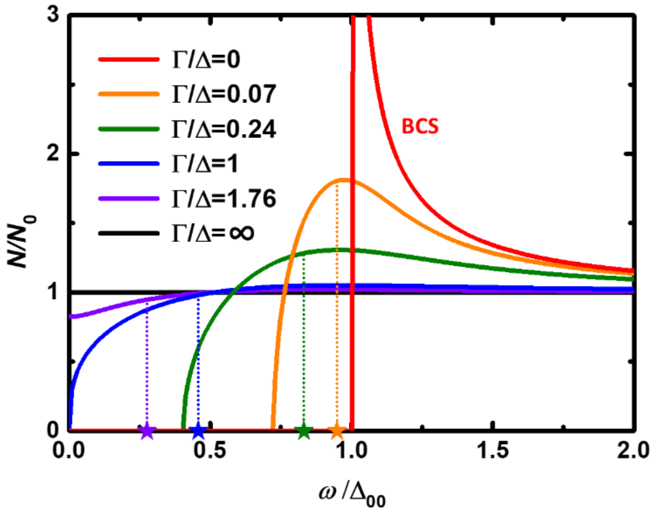

Supplementary Fig. 5 | Density of states of a pair-broken superconductor under an external in-plane magnetic field.

a, The density of states N at $T = 0$ K (normalized to that in the normal state) per one spin plotted as a function of the energy (normalized to the maximum BCS gap in the absence of an external magnetic field) for several values of $\Gamma(H)/\Delta(H)$ as predicted by the Skalski—Betbeder-Maribet—Weiss theory. The solid red line represents BCS theory with zero magnetic field. The blue curve $(\Gamma/\Delta(\Gamma) = 1)$ corresponds to the onset of the gapless regime while the black curve $(\Gamma/\Delta(\Gamma) = \infty)$ corresponds to the normal state. The star symbols denote the values of the superconducting order parameter Δ for each case.

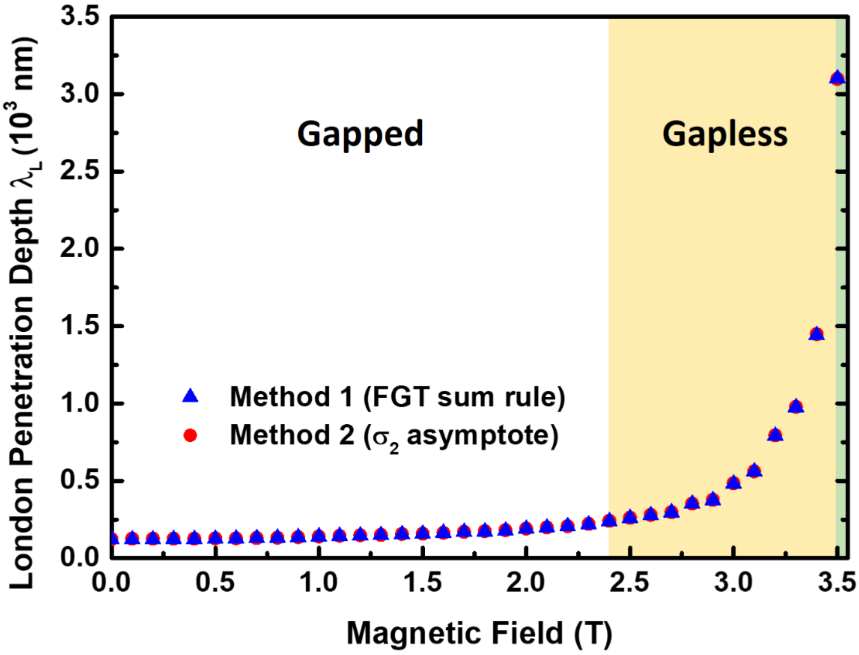

Supplementary Fig. 6 | In-plane magnetic field dependence of the London penetration depth.

London penetration depth is extracted from the Ferrell-Glover-Tinkham (FGT) sum rule (Method 1, blue triangles) and also from the imaginary part of the optical conductivity (Method 2, red circles). Method 1 is based on the conservation of the spectral weight according to the FGT sum rule. In Method 2, the asymptotic form of the imaginary part of the optical conductivity (inversely proportional to frequency) was utilized. The white, yellow, and green shaded regions reveal the gapped superconducting state, the gapless superconducting state, and the normal state.
